# High-Density Genetic Map Construction and Gene Mapping of Basal Branching Habit and Flowers per Leaf Axil in Sesame

**DOI:** 10.3389/fpls.2017.00636

**Published:** 2017-04-27

**Authors:** Hongxian Mei, Yanyang Liu, Zhenwei Du, Ke Wu, Chengqi Cui, Xiaolin Jiang, Haiyang Zhang, Yongzhan Zheng

**Affiliations:** Henan Sesame Research Center, Henan Academy of Agricultural SciencesZhengzhou, China

**Keywords:** *Sesamum indicum* L., SLAF-seq, genetic map, branching habit, flowers per leaf axil

## Abstract

A good genetic map can provide the framework for quantitative trait loci (QTL) analysis, map-based gene cloning, and genome sequence assembling. The main objectives of this study were to develop a high-density genetic linkage map using specific length amplified fragment sequencing (SLAF-seq) in sesame. In the result, a high-resolution genetic map with 9,378 SLAF markers and 13 linkage groups (LGs) was constructed. The map spanned a total genetic distance of 1,974.23 cM, and the mean LG length was 151.86 cM, with an average genetic distance of 0.22 cM between adjacent markers. Based on the newly constructed genetic map, genes for basal branching habit (*SiBH*) and flowers per leaf axil (*SiFA*) were mapped to LG5 and LG11, respectively.

## Introduction

Sesame (*Sesamum indicum* L. 2*n* = 26) belongs to the Pedaliaceae family and is one of the most ancient domestic oil crops (Bedigian and Harlan, 1986). Sesame seed contains about 55% oil, 20% protein, and 13.5% carbohydrates, and is traditionally considered as ‘the queen of oilseeds’ for its high content of unsaturated fatty acids (43% oleic acid, 35% linoleic acid) and rich natural antioxidants such as sesamin and sesamolin (Ashri, 1998; Namiki, 2007). However, its production and extension was limited due to low yields, seed shattering, and high manual labor requirements. At present, sesame is mainly grown in tropical and subtropical regions of Asia, Africa, and Latin America. From 2010 to 2014, the annual acreage of sesame harvested worldwide is about 8.10 × 10^6^ hectares with a total production of 4.67 × 10^6^ metric tons (FAO data), most of which are grown in developing and undeveloped countries, and almost all of them are harvested manually. There is an urgent need to develop new cultivars with high yield potential, resistance/tolerance to biotic and abiotic stresses, and enhanced agronomic traits adapted to mechanized harvest (Uzun et al., 2003; Ashri, 2007; El-Bramawy et al., 2008; Uzun and Cagirgan, 2009).

Branching habit is one of the most important components of the plant architecture, which plays an important role in grain yield and cultivation practices of many crops, including sesame (Baydar, 2005; Teichmann and Muhr, 2015). The branching patterns vary markedly in sesame varieties and germplasm accessions. The wild species have many branches, while there are only few or none branches in landraces and modern cultivars. Number of flowers per leaf axil is another important contributor to sesame yield, and the plants with more flowers have the potential to set more capsules and consequently more seeds. In sesame, there can be one or more (usually three) flowers borne on short pedicels in a leaf axil. When one flower produced, the two lateral primordia differentiate into nectaries. In previous studies, it was indicated that the inheritance of branching habit (branched vs. unbranched) (Nohara, 1933; Brar and Ahuja, 1979; Baydar and Turgut, 2000) and flowers per leaf axil (mono-flower vs. tri-flower) (Nohara, 1933; Sikka and Gupta, 1947) were all determined by one single dominant gene. However, the genetic basis of them has remained elusive.

A genetic map is the prerequisite for mapping and cloning of quantitative trait loci (QTL) or genes for agronomically important traits in crops (Mackay et al., 2009; Würschum, 2012). As an orphan or neglected crop, high-density genetic map in sesame is still limited. The first genetic map of sesame was developed using an F_2_ population with 96 individuals (Wei et al., 2009), and it contained 220 markers, including 8 expressed sequence tag-simple sequence repeats (EST-SSRs), 25 amplified fragment length polymorphism (AFLPs) and 187 Random Selective Amplification of Microsatellite Polymorphic Loci (RSAMPLs), which were distributed on 30 linkage groups (LGs) and spanned 936.72 cM. In 2012, an improved genetic map was constructed using an F_2_ population with 260 plants derived from the same cross and used for mapping QTL of seed coat color (Zhang H. et al., 2013). The linkage map covered 1,216.00 cM, containing 653 marker loci (30 EST-SSR, 50 AFLP, and 573 RSAMPL) distributed on 14 LGs, and 4 QTLs for seed coat color were mapped to LG1, LG11, and LG13, respectively. The advent of next-generation sequencing (NGS) technologies has significantly reduced sequencing load and cost (Davey et al., 2011), especially for several methods combined NGS with restriction enzyme digestion to reduce the complexity of the target genomes, such as reduced-representation sequencing (RRS) using reduced-representation libraries (RRLs) (Van Tassell et al., 2008), restriction-site-associated DNA sequencing (RAD-seq) (Miller et al., 2007), genotyping-by-sequencing (GBS) (Elshire et al., 2011) and specific length amplified fragment sequencing (SLAF-seq) (Sun et al., 2013). The SLAF-seq method has several advantages such as low cost, high efficiency, and accuracy in marker development and genotyping (Sun et al., 2013). It has been widely used for high-density genetic map construction, QTL mapping and genome-wide association studies (GWAS) in soybean (Li et al., 2014; Qi et al., 2014; Zhao et al., 2015), rice (Mao et al., 2015), cotton (Zhang et al., 2016), cucumber (Wei et al., 2014; Xu et al., 2015a,b; Zhu et al., 2016), pumpkin (Zhang et al., 2015), wax gourd (Jiang et al., 2015), tobacco (Gong et al., 2016), and grape (Guo et al., 2015), including sesame (Zhang Y. et al., 2013).

The main objectives of this study were to develop a high-density genetic linkage map using SLAF-seq method and map genes controlling basal branching habit and flowers per leaf axil in sesame.

## Materials and Methods

### Plant Materials and Trait Investigation

A BC_1_ population including 300 individuals was developed from a cross between two sesame cultivars, Yuzhi 4 and Bengal Small-seed (hereinafter BS), with the former as maternal and recurrent parent and the latter as paternal parent. Yuzhi 4 is an elite cultivar developed by Zhumadian Institute of Agricultural Sciences (Henan, China) in 1980s, which had been widely grown in most of the sesame growing regions in China over the last three decades. Meanwhile, Yuzhi 4 is one of the most important founder parents in Chinese sesame breeding programs, from which, more than one third of the cultivars released in the Yellow River and Huaihe River regions had been derived (Liu et al., 2012). BS is an anonymous cultivar introduced from Bangladesh, with multiple basal branches, mono-flower per leaf axil, small seed size, high lignan contents, and late flowering time comparing to Yuzhi 4. Both cultivars have been artificially self-pollinated for at least six generations to ensure pure inbred lines for development of segregating populations. The BC_1_ population, two parents and their F_1_ were grown at Sanya experimental station (N18°14′, E109°29′) of HSRC-HAAS (Hainan, China) in the winter of 2014. Number of branches and flowers per leaf axil were investigated for each plant, and a subset of 150 BC_1_ individuals was randomly selected for genotyping and mapping analysis.

### DNA Extraction and SLAF-Sequencing

Genomic DNA of the two parents and 150 BC_1_ individuals were extracted from young leaves using the CTAB methods as described by Paterson et al. (1999) with some modification to the components of the CTAB buffer (11.69 g NaCl, 2 g CTAB, 2 g PVP40, 5.92 g D-Sorbitol and 1.25 g Na_2_SO_3_ in a total volume of 100 ml of 20 mM EDTA, 100 mM Tris-HCl, pH 8.0) to eliminate ultra-plentiful polysaccharides in sesame leaves. Crude DNA samples were purified using a DNeasy Kit (Qiagen, Valencia, CA, USA), assessed by electrophoresis on 0.8% agarose gel and quantified using spectrophotometry (NanoDrop 8000, Thermo Scientific, USA). The SLAF libraries were constructed following the procedure described by Sun et al. (2013), except that two restriction enzymes HaeIII (recognition site 5′-GG/CC-3′, New England Biolabs, NEB, USA) and Hpy166II (5′-GTN/NAC-3′, NEB) were used to digest the genomic DNA. Pooled samples were separated by 2% agarose gel electrophoresis, and fragments ranging from 264 to 364 base pairs (with indexes and adaptors) in size were excised and purified using a QIAquick gel extraction kit (Qiagen, Hilden, Germany). Gel-purified products were diluted and subjected to pair-end sequencing on an Illumina HiSeq 2000 platform (Illumina Inc., San Diego, CA, USA) at Beijing Biomarker Technologies Corporation^1^.

### SLAF-Seq Data Grouping and Genotyping

The SLAF-seq data grouping and genotyping were performed as described in detail by Sun et al. (2013). Briefly, raw reads were demultiplexed to 152 individuals according to the barcode sequences and reads with quality score lower than 20 were filtered out. High quality reads were clustered based on sequence similarity by BLAT (-tileSize = 10 -stepSize = 5) using one-to-one alignment (Kent, 2002). Identical reads were merged to reduce computational intensity, and sequences with over 90% similarity were grouped into one SLAF locus. Alleles of each SLAF were defined using the minor allele frequency (MAF) evaluation. As a diploid species, sesame can only have at most four alleles at one locus, SLAF groups containing more than four alleles were considered as repetitive and filtered out. SLAFs with 2–4 alleles were identified as polymorphic, and assorted into eight segregation patterns as following: ab×cd, ef×eg, hk×hk, lm×ll, nn×np, aa×bb, ab×cc, and cc×ab. The BC_1_ population used here was derived from two homozygous inbred lines, therefore only the SLAF markers which had segregation patterns of aa×bb were used for subsequent analysis.

### Genetic Map Construction

The high-density linkage map was constructed using the HighMap method (Liu et al., 2014). Briefly, the modified logarithm of odds (mLOD) scores was used to allocate the SLAF markers into LGs and markers with mLOD scores <5 were filtered. SLAF markers in each LG were ordered using the maximum likelihood (ML) algorithm and genotyping errors were corrected with the SMOOTH algorithm. The Kosambi mapping function was employed to convert recombination percentages to genetic distance in cM.

### QTL/Gene Mapping

According to previous studies and our observation, both branching habit (branched vs. unbranched) (Nohara, 1933; Brar and Ahuja, 1979; Baydar and Turgut, 2000) and flowers per leaf axil (mono-flower vs. tri-flower) (Nohara, 1933; Sikka and Gupta, 1947) in sesame are all controlled by one single dominant gene. For flowers per leaf aixl, the phenotype of progenies in segregating populations showed clear binomial distribution (mono-flower or tri-flower), and followed a 1:1 (χ^2^ = 0.03) segregation ratio, indicating that basal branching habit was controlled by one single gene so the trait was treated as a dominant marker and gene mapping was performed by linkage test with SLAF markers. For branching habit, however, the branch number of the BC_1_ individuals showed continuous distribution (0 to 8). Therefore, QTL mapping strategies were employed to scan the target regions controlling branching habit. QTL mapping was performed using the IciMapping 4.1 software (Meng et al., 2015) with the inclusive composite interval mapping additive (ICIM-ADD) model (Li et al., 2007), and LOD threshold was determined by 1,000 permutations (*P* = 0.05).

## Results

### Genotyping the BC_1_ Population Using SLAF-Seq

**FIGURE 1:**
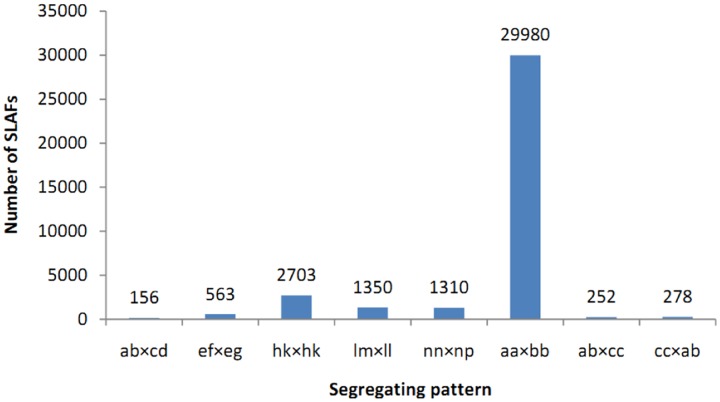
**Number of SLAF markers in each segregation pattern.** The *x*-axis indicates eight segregation patterns grouped by genotype encoding rule and the *y*-axis represents number of SLAF markers.

A total of 71.15 Gb high quality sequence data including 355.78 M 100-bp pair-end reads were generated by high-throughput sequencing of the SLAF library. The average guanine-cytosine (GC) content and Q30 ratio (a quality score of 30) of the data were 39.56 and 87.14%, respectively. According to the index sequences, 12.75, 11.94, and 331.08 M reads were assigned to Yuzhi 4 (maternal and recurrent parent), BS (paternal parent), and the 150 BC_1_ progenies, respectively (**Table 1**). The number of reads for 150 BC_1_ individuals ranged from 892,224 to 2,887,083 with an average of 2,207,207 (**Supplementary Table S1**). High quality pair-end reads with clear index information were clustered based on sequence similarity (Kent, 2002), and a total of 230,620 SLAF tags were obtained after filtering low-depth tags. In parental lines, 208,378 and 196,862 SLAFs were identified for Yuzhi 4 and BS, as well as the average depth were 41.91- and 37.70-fold, respectively (**Table 1**). In the 150 BC_1_ individuals, the number of SLAFs ranged from 123,705 to 166,623 with an average of 149,794, and the mean depth ranged from 4.19- to 15.69-fold with an average of 10.23-fold (**Table 1** and **Supplementary Table S2**). Among the 230,620 SLAFs obtained, 42,115 were polymorphic with a polymorphic rate of 18.26%. The genotypes of two parental lines were designated with different alphabets following a genotype encoding rule to determine segregation patterns, and 36,592 of the 42,115 polymorphic SLAFs were successfully encoded and grouped into eight segregation patterns (ab×cd, ef×eg, hk×hk, lm×ll, nn×np, aa×bb, ab×cc, and cc×ab) (**Figure 1**). Since the two parents (Yuzhi 4 and BS) used here are homozygous inbred lines, and only the 29,980 SLAFs falling into the aa×bb segregation pattern can be used for linkage analysis.

**Table 1:** High quality data generated by sequencing the SLAF library.

Samples	Number of reads	Number of bases	GC contents (%)	Q30 (%)	Number of SLAFs	Average depth
Yuzhi 4	12,754,500	2,550,900,000	39.43	87.19	208,378	41.91
BS	11,940,438	2,388,087,600	39.84	86.27	196,862	37.70
Progenies	331,081,095	66,216,219,000	39.40	87.97	149,794	10.23
Total	355,776,033	71,155,206,600	39.56	87.14	230,620	–


### High-Density Genetic Map Constructed with SLAF Markers

**FIGURE 2:**
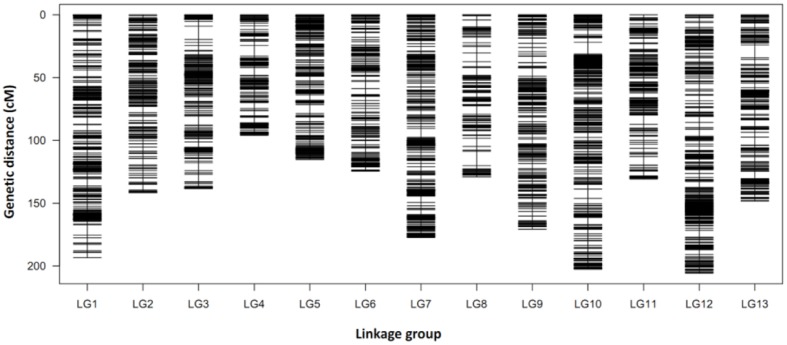
**Distribution of SLAF markers on 13 LGs of sesame.** A black bar indicates a marker. The *x*-axis indicates LG and the *y*-axis represents genetic distance (cM as unit).

To ensure the accuracy of genotyping, the following steps were performed: (1) SLAFs with depth of less than 10-fold in each parent were discarded, (2) SLAFs with more than three SNPs were removed, (3) SLAFs with depth of less than twofold in each of the 150 progenies were defined as missing data, and (4) SLAFs with more than 30% missing data or with excessively distorted segregation ratios (χ^2^ test, *P* ≤ 0.01) were excluded. Therefore, a final set of 9,823 high quality SLAF markers were used for genetic map construction using the HighMap method (Liu et al., 2014). As a result, a high-density genetic map with 13 LG and 9,378 SLAFs was constructed (**Supplementary Table S3** and **Data S1**). The map covered a genetic distance of 1,974.23 cM in total, with an average distance of 0.22 cM between adjacent markers. The length of each LG ranged from 96.02 cM (LG4) to 205.97 cM (LG12) with an average of 151.86 cM, and the number of SLAFs per LG varied from 497 (LG6) to 1,196 (LG12) with an average of 721. Three large gaps more than 10 cM located on LG3 (10.47 cM), LG8 (11.29 cM) and LG13 (10.17 cM), respectively (**Table 2**, **Figure 2** and **Supplementary Table S3**).

**Table 2:** Summary of the high-density genetic map constructed in sesame.

Linkage group	Marker number	Length (cM)	Average distance (cM)	Largest gap (cM)	SNP number
LG1	855	193.34	0.23	8.13	1,241
LG2	526	141.72	0.27	4.96	757
LG3	700	138.34	0.20	10.47	1,040
LG4	498	96.02	0.19	8.41	806
LG5	725	115.27	0.16	7.28	1,109
LG6	497	124.45	0.25	5.60	706
LG7	914	177.31	0.19	7.18	1,320
LG8	612	129.12	0.21	11.29	931
LG9	652	170.87	0.26	6.90	1,018
LG10	1,085	202.59	0.19	9.39	1,726
LG11	531	130.84	0.25	7.56	799
LG12	1,196	205.97	0.17	5.45	1,832
LG13	587	148.39	0.25	10.17	855
Total	9,378	1,974.23	0.22	–	1,4170


### QTL/Gene Mapping of Basal Branching Habit and Flowers Per Leaf Axil

**FIGURE 3:**
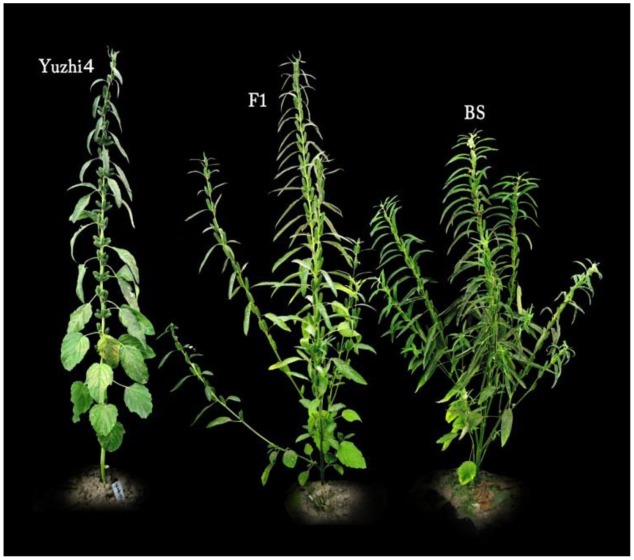
**Phenotype of basal branching habit and flowers per leaf axil of Yuzhi 4, BS and their F_1_.** Yuzhi 4: unbranched and tri-flower; F_1_: branched and mono-flower; BS: branched and mono-flower.

Yuzhi 4 is a cultivar with uniculm (unbranched type) and tri-flower per leaf axil, while BS is a line with branched type and mono-flower per axil. The F_1_ plants all showed branched and mono-flower phenotype, which confirmed the dominant nature of gene action for mono-flower to tri-flower and branched to unbranched types (**Figure 3**).

**FIGURE 4:**
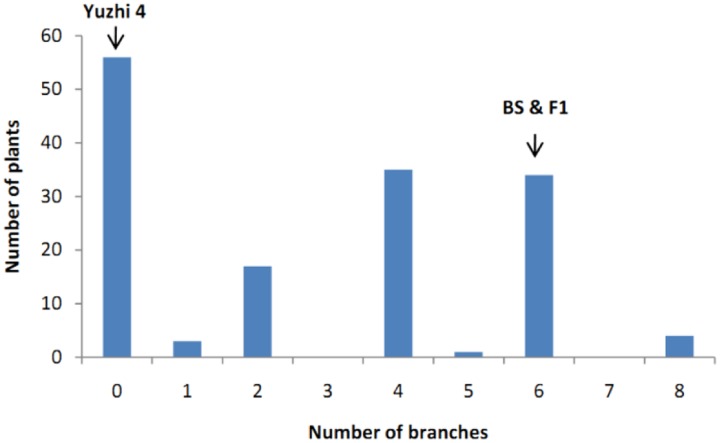
**Distribution of number of branches in 150 BC_1_ progenies.** The *x*-axis indicates number of branches and the *y*-axis is the number of plants with different number of branches.

**FIGURE 5:**
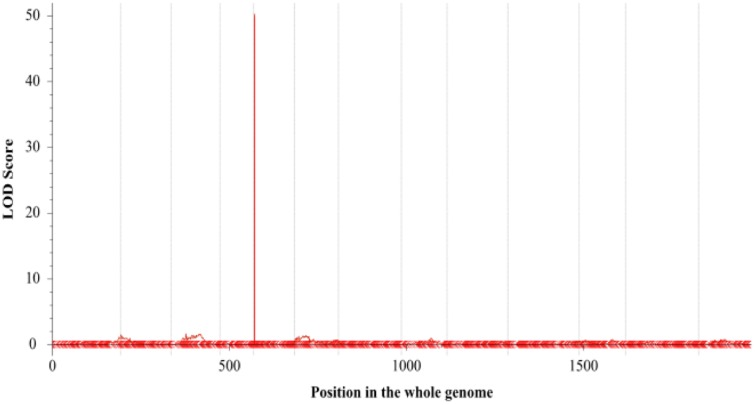
**Quantitative trait loci (QTL) analysis of number of branches using the high-density genetic map.** The *x*-axis indicates map position (cM) across the 13 LGs and the *y*-axis is LOD score.

**FIGURE 6:**
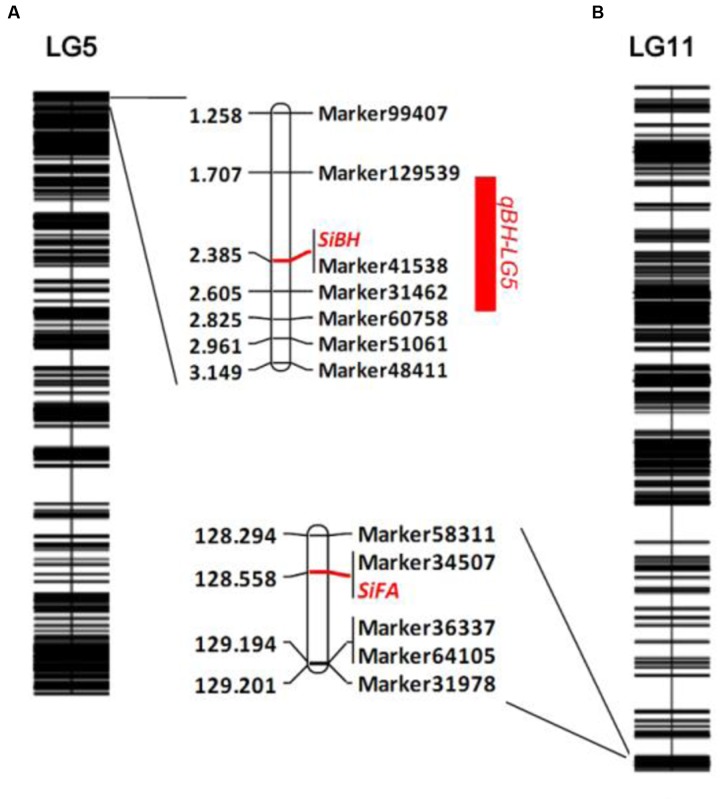
**Gene mapping of basal branching habit and flowers per leaf axil in sesame.** **(A)** QTL analysis of number of branches and gene mapping branching habit. **(B)** Gene mapping of flowers per leaf axil.

The number of branches of 150 BC_1_ plants showed continuous distribution with a range of 0–8 (**Figure 4**). At first, QTL analysis was performed using the software QTL IciMapping 4.1 with ICIM-ADD model (Li et al., 2007). In this way, a major QTL (designated as *qBH-LG5*) with LOD score of 50.27 was detected on the LG 5 at the position of 2.50 cM, neighbored by Marker41538 and Marker31462 with a confidence interval of 1.75 to 2.75 cM, which explained 78.64% of the phenotypic variation (PVE) (**Figures 5**, **6A**). If plants with branches ≤2 were classified as “unbranched type” and ≥3 as “branched type,” the branched and unbranched plants in the BC_1_ populations were 76 and 74, respectively, which followed a 1:1 (χ^2^ = 0.03) segregation ratio, indicating that basal branching habit was controlled by one single gene (designated as *SiBH*). Interestingly, the *SiBH* gene was mapped to LG5 at 2.385 cM by linkage analysis, and the position was almost as same as that of *qBH-LG5*. Three markers (Marker129539, Marker41538, and Marker31462) were found to be tightly linked. Among them, marker41538 was co-segregated with *SiBH* and the genetic distance of Marker129539 and Marker31462 in the flanking regions were 0.68 and 0.22 cM, respectively (**Figure 6A**). For flowers per leaf axil, 85 of the 150 BC_1_ individuals showed mono-flower and 65 showed triple-flower per axil (χ^2^ = 2.67 for 1:1 ratio), which confirmed that flowers per axil was controlled by one single gene (designated as *SiFA*). In the high-density map, the *SiFA* gene was mapped to LG11 at 128.56 cM, and three markers (Marker58311, Marker34507, and Marker36337) were found to be tightly linked. Among them, marker34507 was co-segregated with *SiFA*, and the genetic distance of Marker58311 and Marker36337 in the flanking regions were 0.26 and 0.64 cM, respectively (**Figure 6B**).

## Discussion

### High-Density Genetic Map Constructed in Sesame

The first high-density genetic map of sesame was developed using the SLAF-seq method with an F_2_ population including 107 individuals (Zhang Y. et al., 2013). The map contained 1,233 SLAF markers on 15 LGs, and covered 1,474.87 cM in total length, with an average distance of 1.20 cM. Wu et al. (2014) constructed a genetic map using the RAD-seq method with a population of 224 recombinant inbred line (RIL) lines. The map was consisted of 1,230 markers distributing on 14 LGs and spanned 844.46 cM in length with an average of 0.69 cM between adjacent markers. Using this map and RIL population, they detected 13 QTLs on 7 LGs and 17 QTLs on 10 LGs for seven yield-related traits by the multiple interval mapping (MIM) and the mixed linear composite interval mapping (MCIM), respectively. In this study, we constructed a high-density genetic map using the SLAF-seq method with a BC_1_ population including 150 individuals. This map comprised 9,378 SLAF markers on 13 LGs, and covered a genetic distance of 1,974.23 cM in total, with an average distance of 0.22 cM between adjacent markers (**Figure 2** and **Supplementary Table S3**). Compared to previously developed high-density genetic maps in sesame, the map constructed in this study had the highest marker density, fewer and smaller gaps, and equal number of LGs to *Sesamum indicum* L. chromosomes (2*n* = 26) (**Table 3**).

**Table 3:** Comparison of three high-density genetic maps developed with reduced-representation sequencing in sesame.

Reference	Population	Sample size	Method	Linkage group No.	Marker No.	Total length (cM)	Average distance (cM)	Largest Gap (cM)	Gaps ≥10 cM
Zhang Y. et al., 2013	F_2_	107	SLAF-seq	15	1,233	1474.87	0.23	29.10	9
Wu et al., 2014	RIL	224	Rad-seq	14	1,230	844.46	0.69	22.54	16
This study	BC_1_	150	SLAF-seq	13	9,378	1,974.23	0.22	11.29	3


Wang et al. (2016) sequenced 430 RILs using the RAD-seq method and developed a bin map by joining the consecutive intervals on the genome that lacked a recombination event within the population. The map included 1,522 bins on 13 LGs (SLGs), with a total length of 1090.99 cM and a mean distance of 0.72 cM between adjacent bins. The map was used to improve Zhongzhi 13 genome assembly from version 1.0 to version 2.0. This map was the first one developed with large population in sesame. In theory, large population size allows precisely estimate recombination events between linked markers and ultimately determines map resolution and accuracy (Ferreira et al., 2006). In spite of this, there still were five scaffolds with lengths greater than 150 kb unanchored in the improved Zhongzhi 13 genome assembly version 2.0 (Wang et al., 2016). This may be caused by low genetic dissimilarity between the two parents or these scaffolds containing high repetitive sequence. Since the bin map strategy was adopted in their study, the properties of the map were not easy to compare with that of the map constructed in our study. In our study, among the 230,620 SLAF markers obtained, 42,115 were polymorphic with a polymorphic rate of 18.26%, which was higher than that reported by Zhang Y. et al. (2013) and Wu et al. (2014). Even if the redundant markers (markers on the same locus) are merged, there still are 4,006 loci in our genetic map (**Supplementary Table S3**). Although redundant markers cannot provide additional information, we have showed them in the current high-density genetic map and hope that it may serve as another framework for QTL mapping and genome assembly improvement.

### Inheritance of Branching Habit and Flowers Per Leaf Axil in Sesame

Branching habit can influence seed yield and cultivation practices, which will play an important role in development of cultivars suitable for mechanized harvest in sesame production. Nohara (1933) reported that the branching habit was dominant, and suggested a single gene difference but also commented about the difficulty in classification due to fluctuation brought about by environment. Brar and Ahuja (1979) reported that the branching habit was monogenically controlled, and the monostem (unbranched) characteristic was controlled by recessive gene. Baydar and Turgut (2000) observed 3:1 segregation ratio for branching and non-branching types. However, branching habit are often influenced by environmental factors. For example, high density planting can suppress branch number of branched types, and even unbranched type can produced more than one branches in low density populations. Other environmental factors influencing branching habit include photoperiod, light levels and quality, plant nutrition status, and availability of nutrients. Factors mentioned above usually lead to a continuous distribution of number of branches between the branched and unbranched types, the plants at the edges can be easily identified, but the ones in the middle are difficult to classify. Wang et al. (2009) performed both linkage test and QTL mapping to map a Fusarium wilt resistant gene in upland cotton, and a major QTL and the *FW^R^* gene were both detected in the same genomic region. The development of Fusarium wilt disease symptom in cotton is affected by several environmental factors, which is similar to branch development in sesame. Therefore, both linkage analysis and QTL mapping strategies were employed to mapping the target genes in this study. In the results, a single major QTL *qBH-LG5* with LOD score of 50.27 and 78.64% of the PVE explanation was identified on LG5 at position of 2.50 cM by QTL mapping, as well as the *SiBH* gene was mapped to LG5 at 2.385 cM by linkage analysis and the position was almost as same as that of *qBH-LG5* (**Figure 6A**). It is noteworthy that, in spite of single gene inheritance for branching habit were found in the current and previous studies, the branching patterns in sesame are more complex. The International Plant Genetic Resources Institute (IPGRI) described branching habit into non-branching, basal-branching, top-branching, and other types in sesame germplasm characterization (IBPGR, 2004), and the International Union for the Protection of New Varieties of Plants (UPOV) classified branching patterns into absent or very few, medium and very many types according to number of branches, and into basal branching, branching along stem and apical branching types according to position of branches in distinctness, uniformity, and stability (DUS) test for protection of intellectual property of new varieties^2^. The branching patterns mentioned above were all found in the characterization of a 5200-accession germplasm collection conserved in our library. Whether if basal, top branching and branching along stem types are all controlled by one single gene, or the genes are allelic, and genetic basis of medium and very many branch-types are needed further detailed research.

Comparing to branching habit, the inheritance of number of flowers per leaf axil is much simple. It was indicated by previous studies that flowers per leaf axil was controlled by one single gene with dominant effect of mono-flower vs. tri-flower (Nohara, 1933; Sikka and Gupta, 1947). In this study, the *SiFA* gene behavior and genetic effect were validated and mapped to LG11 at 128.56 cM. The tightly linked markers for basal branching habit (Marker129539, Marker41538, and Marker31462) and flowers per leaf axil (Marker58311, Marker34507, and Marker36337) could provide useful information for further MAS breeding and map-based gene cloning studies.

## Conclusion

In this study, a total of 71.15 Gb high quality sequence data were generated by high-throughput sequencing of the SLAF libraries, and a high-resolution genetic map with 9,378 SLAF markers was developed. The map comprised 13 LGs, which equaled the number of sesame chromosomes, spanned a total genomic distance of 1,974.23 cM, and the mean LG length was 151.86 cM, with an average genetic distance of 0.22 cM between adjacent markers. Based on the map, genes for basal branching habit (*SiBH*) and flowers per leaf axil (*SiFA*) were mapped to LG5 and LG11, respectively. The results will not only provide a platform for QTL/gene fine mapping, map-based gene cloning, and molecular breeding for sesame, but also provide a reference linkage map to help anchor sequence scaffolds of the physical map in improvement of genome assembly.

## Author Contributions

HM and ZD constructed the BC_1_ population. HM and YL performed data analysis and linkage mapping. HM wrote and YL revised the manuscript. KW, CC, and XJ performed some of the field work and took part in DNA extraction. HZ provided valuable research ideas. YZ designed and supervised the study. All authors read and approved the final manuscript.

## Conflict of Interest Statement

The authors declare that the research was conducted in the absence of any commercial or financial relationships that could be construed as a potential conflict of interest.

## Footnotes

1 http://Biomarker.com.cn/

2 http://www.upov.int/resource/en/dus_guidance.html

## Supplementary Material

**TABLE S1 | Summary of sequence data generated by SLAF-seq in 150 BC_1_ individuals.**

**TABLE S2 | Summary information of SLAF markers identified in 150 BC_1_ individuals.**

**TABLE S3 | Map position of 9,378 mapped SLAF markers.**

**DATA S1 | Sequence of 9,378 mapped SLAF markers.**
